# Pathologically Confirmed Dual Coronavirus Disease 2019-Associated Tracheobronchial Aspergillosis and Pulmonary Mucormycosis in a Non-Endemic Region: A Case Report

**DOI:** 10.3390/jcm14155526

**Published:** 2025-08-05

**Authors:** Keon Oh, Sung-Yeon Cho, Dong-Gun Lee, Dukhee Nho, Dong Young Kim, Hye Min Kweon, Minseung Song, Raeseok Lee

**Affiliations:** 1 Vaccine Bio Research Institute, College of Medicine, The Catholic University of Korea, Seoul 06591, Republic of Korea; oh_geon@catholic.ac.kr (K.O.); cho.sy@catholic.ac.kr (S.-Y.C.); symonlee@catholic.ac.kr (D.-G.L.); nhodh@catholic.ac.kr (D.N.); kimpengking2@gmail.com (D.Y.K.); aquaviolet88@naver.com (H.M.K.); enea3587@gmail.com (M.S.); 2 Division of Infectious Diseases, Department of Internal Medicine, College of Medicine, The Catholic University of Korea, Seoul 06591, Republic of Korea

**Keywords:** COVID-19, mucormycosis, aspergillosis, hematopoietic stem cell transplantation, case reports

## Abstract

**Background:** Coronavirus disease 2019 (COVID-19) has led to the expansion of the spectrum of invasive fungal infections beyond traditional immunocompromised populations. Although COVID-19-associated pulmonary aspergillosis is increasingly being recognised, COVID-19-associated mucormycosis remains rare, particularly in non-endemic regions. Concurrent COVID-19-associated invasive tracheobronchial aspergillosis and pulmonary mucormycosis with histopathological confirmation is exceedingly uncommon and poses significant diagnostic and therapeutic challenges. **Case presentation:** We report the case of a 57-year-old female with myelodysplastic syndrome who underwent haploidentical allogeneic haematopoietic stem cell transplantation. During post-transplant recovery, she developed COVID-19 pneumonia, complicated by respiratory deterioration and radiological findings, including a reverse halo sign. Bronchoscopy revealed multiple whitish plaques in the right main bronchus. Despite negative serum and bronchoalveolar lavage fluid galactomannan assay results, cytopathological examination revealed septate hyphae and *Aspergillus fumigatus* was subsequently identified. Given the patient’s risk factors and clinical features, liposomal amphotericin B therapy was initiated. Subsequent surgical resection and histopathological analysis confirmed the presence of *Rhizopus microsporus*. Following antifungal therapy and surgical intervention, the patient recovered and was discharged in stable condition. **Conclusions:** This case highlights the critical need for heightened clinical suspicion of combined invasive fungal infections in severely immunocompromised patients with COVID-19, even in non-endemic regions for mucormycosis. Early tissue-based diagnostic interventions and prompt initiation of optimal antifungal therapy are essential for obtaining ideal outcomes when co-infection is suspected.

## 1. Introduction

Invasive fungal infections (IFIs) have traditionally represented life-threatening complications in immunocompromised populations, such as those with haematological malignancies or organ transplants. However, growing global reports of influenza-associated pulmonary aspergillosis [1,2,3,4] and coronavirus disease 2019 (COVID-19)-associated pulmonary aspergillosis (CAPA) [5,6] suggest that IFIs may also complicate severe viral respiratory infections, even in the absence of classical host factors. With an intensive care unit prevalence of approximately 10% and mortality rates exceeding 50% in several studies [5,7,8], CAPA has emerged as a serious complication with a high risk of fatality.

Reports of COVID-19-associated mucormycosis (CAM) have emerged primarily from mucormycosis-endemic regions, particularly in patients with diabetes mellitus [9]. Unlike invasive aspergillosis, the diagnosis of mucormycosis is hindered by the lack of reliable indirect microbiological evidence or biomarkers, and the disease is associated with extremely poor outcomes. More recently, several cases of co-infection with *Aspergillus* spp. and *Mucorales* have been reported in mucormycosis-endemic regions [10,11], especially among critically ill or severely immunocompromised patients, highlighting the challenges of timely recognition and management.

Herein, we report a case of COVID-19-associated tracheobronchial aspergillosis (TBA) and pulmonary mucormycosis co-infection in a haematopoietic stem cell transplantation (HSCT) recipient in South Korea—a non-endemic region where such dual infections are exceptionally rare. The diagnosis was confirmed through histopathological examination following a proactive diagnostic approach.

## 2. Case Presentation

A 57-year-old female with myelodysplastic syndrome (MDS-IB-2 with a TP53 [17p13.1] mutation) underwent allogeneic HSCT from a haploidentical sibling donor (3/6 human leukocyte antigen match) after achieving complete remission with decitabine. Her medical history and findings on physical examination were otherwise unremarkable. Pre-transplant laboratory workup showed the following results: ferritin, 2350 ng/mL; haemoglobin, 7.0 g/dL; absolute neutrophil count, 420/μL; and positive cytomegalovirus serology. Chest radiography, echocardiography, upper gastrointestinal endoscopy, and abdominal ultrasonography findings were unremarkable.

The conditioning regimen included fludarabine, busulfan, anti-thymocyte globulin (2 mg/kg on days −3 and −2), methylprednisolone (125 mg/day for 2 days), and total body irradiation (400 cGy at day −1). Graft-versus-host disease prophylaxis consisted of post-transplant cyclophosphamide (days +3 and +4), mycophenolate mofetil, and tacrolimus. Prophylactic antimicrobials included ciprofloxacin, micafungin, acyclovir, and letermovir.

On post-transplant day (PTD) 8, the patient developed hypoxia and required supplemental oxygen therapy. SARS-CoV-2 infection was confirmed by RT-PCR. Chest CT revealed bilateral ground-glass opacities, indicative of COVID-19 pneumonia. She received remdesivir (for 5 days) and dexamethasone (6 mg daily). Serum galactomannan and cytomegalovirus RQ-PCR results were negative.

**Figure 1:**
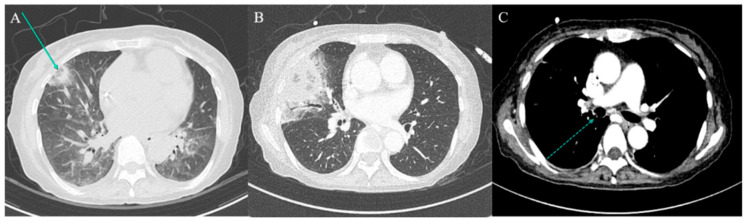
Serial chest computed tomography (CT) findings. (**A**) Low-dose chest CT performed on post-transplant day (PTD) 15 due to persistent haziness on chest radiograph reveals multiple nodular consolidations, including a reverse halo sign (arrow) in the right middle lobe (RML). (**B**,**C**) Contrast-enhanced chest CT on PTD 28 shows a new luminal nodular lesion in the right main bronchus (dotted arrow) and progression of the previously noted RML consolidation.

On PTD 14, she reported right-sided pleuritic chest pain. Repeat CT revealed ground-glass opacity with surrounding consolidation in the right middle lobe, suggestive of a reverse halo sign (Figure 1A). Laboratory investigations performed at this point showed an absolute neutrophil count of 450/μL, negative serum galactomannan result, serum ferritin level of 2845 mg/mL, HbA1c level of 6.1%, and glycoalbumin level of 19.2% (reference range, 11 to 16). On PTD 20, the lesion progressed, prompting initiation of methylprednisolone (45 mg, 1 mg/kg) due to suspected bronchiolitis obliterans organising pneumonia (Supplementary File S1: Figure S1).

**Figure 2:**
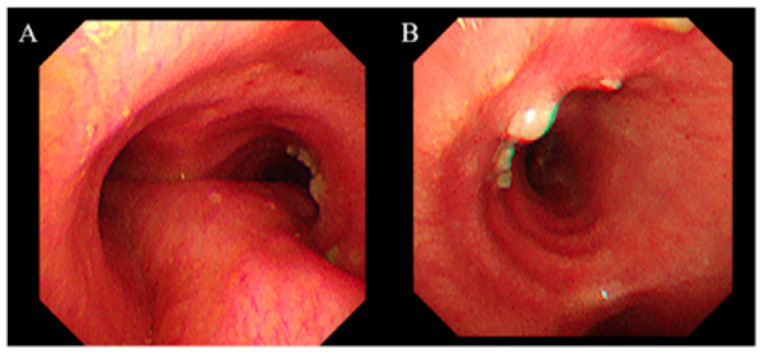
Bronchoscopic findings of the right main bronchus. Bronchoscopy performed on post-transplant day 36 shows multiple white plaques on the right main bronchial mucosa. (**A**) View from the carina. (**B**) View of the right main bronchus. Tissue samples were obtained via lesion biopsy and bronchoalveolar lavage from the right middle lobe.

**Figure 3:**
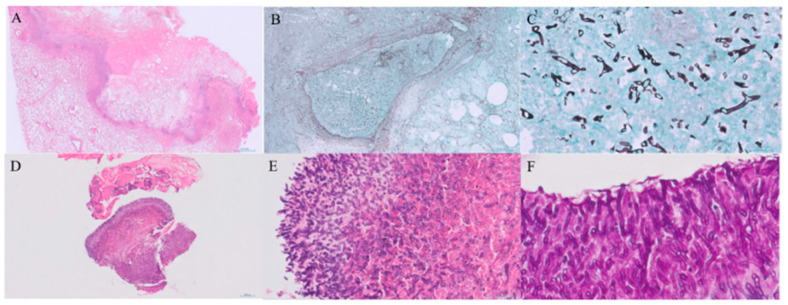
Histopathological findings of invasive mucormycosis and tracheobronchial aspergillosis. (**A**) Low-power view of a haematoxylin and eosin (H&E)-stained section of the resected lung (scale bar = 2.0 mm). (**B**) Grocott–Gomori methenamine silver (GMS) stain showing vascular invasion by fungal hyphae (scale bar = 500 μm). (**C**) High-power GMS-stained section demonstrating broad, non-septate hyphae consistent with *Mucorales* (scale bar = 50 μm). (**D**,**E**) H&E-stained sections of the bronchial biopsy showing septate hyphae with acute-angle branching (scale bars = 200 μm and 50 μm, respectively). (**F**) Periodic acid–Schiff (PAS) staining highlights septate hyphae with dichotomous branching, characteristic of *Aspergillus* spp. (scale bar = 20 μm).

On PTD 28, contrast-enhanced CT revealed worsening of the right middle lobe lesion and a new nodular endobronchial lesion in the right main bronchus (Figure 1C). Bronchoscopy revealed multiple whitish elevated lesions (Figure 2A,B). Cytopathological examination of bronchoalveolar lavage (BAL) fluid revealed septate hyphae with acute-angle branching, suggestive of *Aspergillus* species, although BAL galactomannan was negative. A presumptive diagnosis of COVID-19-associated TBA and pulmonary mucormycosis was established [5]. Liposomal amphotericin B (5 mg/kg/day) was initiated, and the dose of corticosteroids was tapered. On PTD 37, right middle lobectomy with wedge resection of the right upper lobe was performed for diagnostic confirmation and source control. Histopathological examination confirmed invasive mucormycosis (Figure 3A–C) and *Rhizopus microsporus* was cultured. Concurrently, biopsy of the right bronchial lesion revealed septate hyphae, and *Aspergillus fumigatus* complex was isolated, as shown in Figure 3D–F. Thus, the final diagnosis of pathologically confirmed COVID-19-associated TBA and pulmonary mucormycosis was established [5].

The patient’s condition gradually improved, and antifungal therapy was transitioned to oral isavuconazole (200 mg thrice daily for 2 days, then 200 mg daily) on PTD 56. The patient was discharged on PTD 59 in stable condition; a subsequent bone marrow biopsy confirmed that she achieved complete morphologic remission and sustained full donor chimerism. The patient’s detailed clinical course is summarised in Supplementary File S1: Figure S2.

## 3. Discussion and Conclusions

We report an exceptionally rare case of histopathologically confirmed concurrent COVID-19-associated TBA and pulmonary mucormycosis in an HSCT recipient in South Korea, a non-endemic region for mucormycosis. Although CAPA has been increasingly recognised as a complication of severe COVID-19 pneumonia, reports of CAM, particularly simultaneous CAPA and CAM, remain exceedingly rare. In this case, coinfection was initially suspected based on the reverse halo sign on chest CT and bronchoscopic findings of whitish plaques. Histopathological confirmation from both bronchial biopsy and surgical lung resection specimens underscores the importance of early, tissue-based diagnostic efforts.

The spectrum of IFIs, traditionally confined to immunocompromised hosts, has expanded to critically ill patients without conventional host factors [12]. However, severe respiratory viral infections, such as COVID-19, can damage the respiratory epithelium and provoke a placing even individuals without classical host factors (e.g., corticosteroid therapy, deferoxamine use, haematological malignancy, or receipt of HSCT or solid-organ transplant) at risk for IFIs [5,13,14,15]. Many reports of concurrent CAPA and CAM originate from endemic areas, such as India and Iran, where rhino-orbito-cerebral mucormycosis predominates [11]. In contrast, pulmonary mucormycosis is more frequently observed outside endemic regions [11]. Among *Mucorales*, *Rhizopus microsporus* is the second most frequently isolated, and *Aspergillus fumigatus* remains the leading cause of aspergillosis. Although the dual culture of *Aspergillus* species and *Mucorales* occurs in approximately 60% of reported cases, simultaneous histopathological confirmation of both moulds is uncommon [11,16]. To date, only one other histologically proven case of concurrent CAPA and CAM has been reported in South Korea [17]. In this case, coinfection was recognised only after clinical deterioration despite appropriate antifungal therapy targeting *Aspergillus* species, highlighting the diagnostic challenges posed by overlapping features.

Mixed fungal infections represent a serious complication in patients with haematologic malignancies, while comprising a minority of all IFIs [18]. Upon a classical risk factor study of IFIs, a retrospective study from a tertiary centre found systemic corticosteroid use as a risk factor for developing mixed fungal infection [19]. Given the high burden of IFI risk factors, among patients with haematologic malignancies, clinicians must employ all available diagnostic modalities, maintain a high index of suspicion for more than one pathogen, and endeavour to obtain appropriate specimens―including tissue samples―to ensure prompt and accurate identification of mixed fungal infections.

In this patient, COVID-19-associated TBA was initially suspected based on bronchoscopic findings [5]. However, negative serum and BAL galactomannan results, the presence of a reverse halo sign on chest CT, and a history of high-dose corticosteroid exposure—administered during the pre-transplant conditioning regimen and later for presumed bronchiolitis obliterans organising pneumonia—collectively raised concern for mucormycosis co-infection. The reverse halo sign on chest CT―a central ground-glass opacity surrounded by consolidation―played a crucial role in this patient’s diagnosis and can serve as an early radiographic clue to pulmonary mucormycosis, having been observed in 94% of neutropenic leukaemia patients with proven pulmonary mucormycosis versus <1% of those with invasive aspergillosis [20]. Consequently, liposomal amphotericin B was initiated instead of voriconazole. A major challenge in managing invasive fungal infections in the context of COVID-19 is the overlapping clinical and radiological features with COVID-19 pneumonia itself, compounded by limitations in performing invasive procedures amid infection control concerns [5,14]. Non-invasive biomarkers such as serum galactomannan and beta-D-glucan, and BAL galactomannan play a central role in CAPA but lack a diagnostic role for CAM [14]. In patients with clinical suspicion of IFIs who have negative serum and BAL galactomannan or exhibit radiologic features characteristic of mucormycosis, definitive diagnostic interventions—such as repeat bronchoscopy and, where appropriate, surgical resection—are essential to establishing a conclusive diagnosis. Furthermore, emerging molecular diagnostics offer promise to bridge the gap between clinical suspicion and definitive diagnosis. These include PCR assays on serum, BAL fluid, and urine, multiplex qPCR panels simultaneously detecting multiple *Mucorales* genera, and metagenomic next-generation sequencing of clinical specimens―all demonstrating promising sensitivity and specificity for early mucormycosis detection [21,22]. Moreover, as voriconazole lacks activity against *Mucorales*, the early selection of optimal antifungal agents, such as liposomal amphotericin B or isavuconazole, may be crucial for effective treatment [23].

In conclusion, concurrent COVID-19-associated TBA and CAM remains an exceptionally rare but critical clinical entity, even in non-endemic areas. In severely immunocompromised patients with COVID-19 pneumonia, clinicians should maintain a high index of suspicion for dual fungal infections, especially when the clinical or radiological findings are atypical or when there is a poor response to standard antifungal therapy. Early consideration of mucormycosis co-infection—especially in patients with corticosteroid exposure—and timely selection of appropriate antifungal therapy are pivotal for improving outcomes.

## Data Availability

The original contributions presented in this study are included in the article/Supplementary Materials. Further inquiries can be directed to the corresponding author.

## Supplementary Materials

The following supporting information can be downloaded at: https://www.mdpi.com/article/10.3390/jcm14155526/s1, Supplementary Materials File S1: Figure S1: Corticosteroid administration and serial chest radiographs between post-transplant day 12 and day 22; Figure S2: Clinical course of the patient. Supplementary Materials File S2: CAREchecklists.

## Abbreviations

BAL	Bronchoalveolar lavage
CAM	COVID-19-associated mucormycosis
CAPA	COVID-19-associated pulmonary aspergillosis
COVID-19	Coronavirus disease 2019
CT	Computed tomography
HSCT	Haematopoietic stem cell transplantation
IFI	Invasive fungal infection
MDS-IB-2	Myelodysplastic syndrome with increased blasts 2
PTD	Post-transplant day
TBA	Tracheobronchial aspergillosis
TP53	Tumour protein p53
PCR	Polymerase chain reaction
qPCR	Quantitative polymerase chain reaction
RT-PCR	Reverse transcription-polymerase chain reaction
RQ-PCR	Real-time quantitative polymerase chain reaction
SARS-CoV-2	severe acute respiratory syndrome coronavirus 2
