# Supplementary material for: Pathologically Confirmed Dual Coronavirus Disease 2019-Associated Tracheobronchial Aspergillosis and Pulmonary Mucormycosis in a Non-Endemic Region: A Case Report
Source: J Clin Med. 2025 Aug 5;14(15):5526. doi: 10.3390/jcm14155526 (PMC12347539; doi:10.3390/jcm14155526)
Supplement: Supplementary file 1 [file jcm-14-05526-s001.zip › jcm-3751502-supplementary/Supplementary_materials_S1.pdf]

Supplementary materials

Table of contents

Supplementary Figure S1. Corticosteroid administration and serial chest radiographs between post-transplant day 12 and day 22 2

Supplementary Figure S2. Clinical course of the patient 4

Supplementary figure S1. Corticosteroid administration and serial chest radiographs between post-transplant day 12 and day 22.

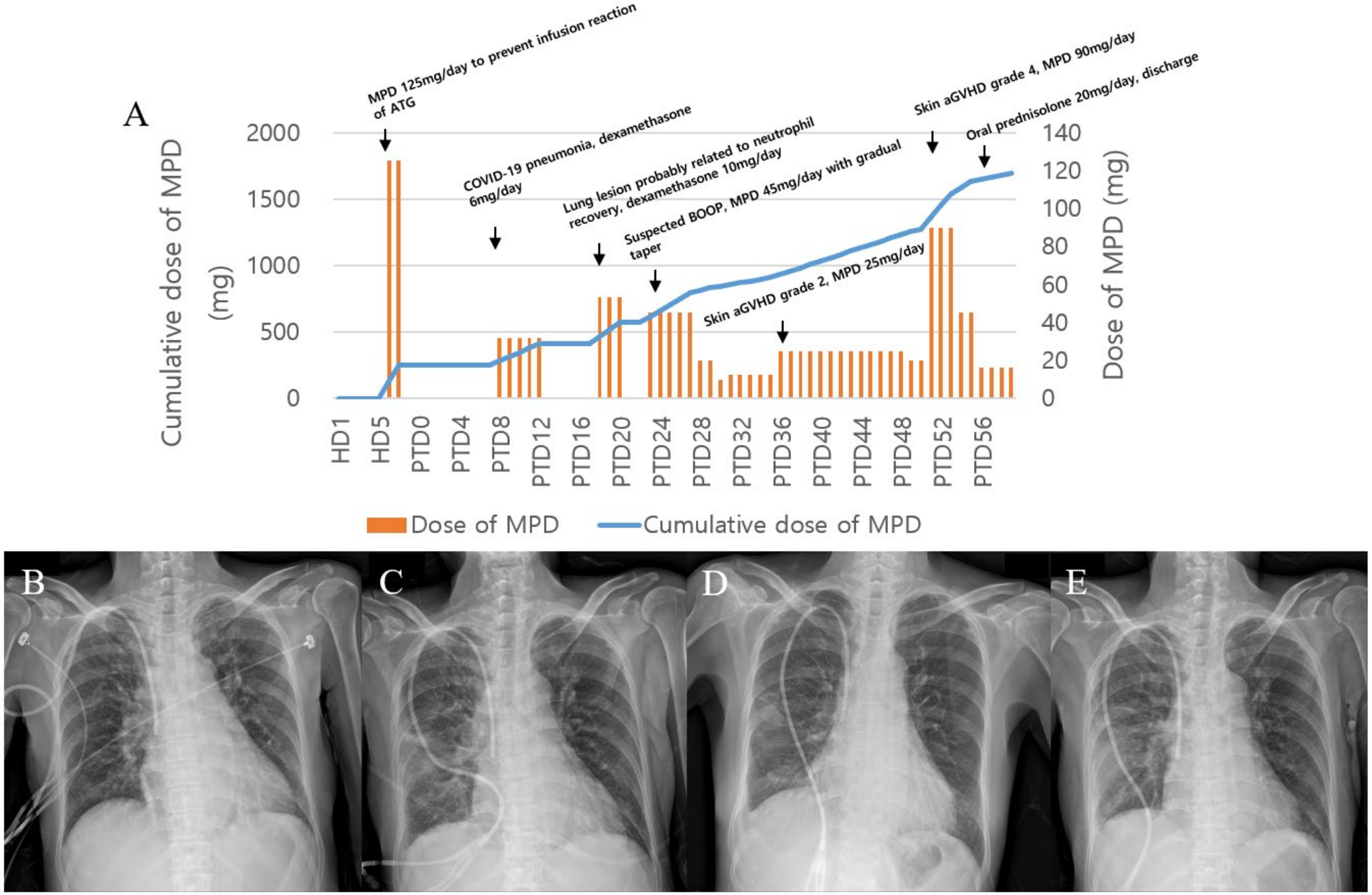

(A) Timeline of corticosteroid administration and cumulative methylprednisolone-equivalent dose. During hospitalisation, methylprednisolone, dexamethasone, and prednisolone were administered. Dexamethasone (0.75 mg) and prednisolone (5 mg) were each converted to methylprednisolone equivalent of 4 mg. (B-E) Chest radiographs on post-transplant days 12, 18, 20, and 22, respectively. The right middle lung field lesion initially regressed following dexamethasone administration but progressed again. aGVHD, acute graft-versus-host disease; ATG, anti-thymocyte globulin; BOOP, bronchiolitis obliterans organising pneumonia; COVID-19, coronavirus disease 2019; HD, hospital day; MPD, methylprednisolone; PTD, post-transplant date.

Supplementary Figure S2. Clinical course of the patient

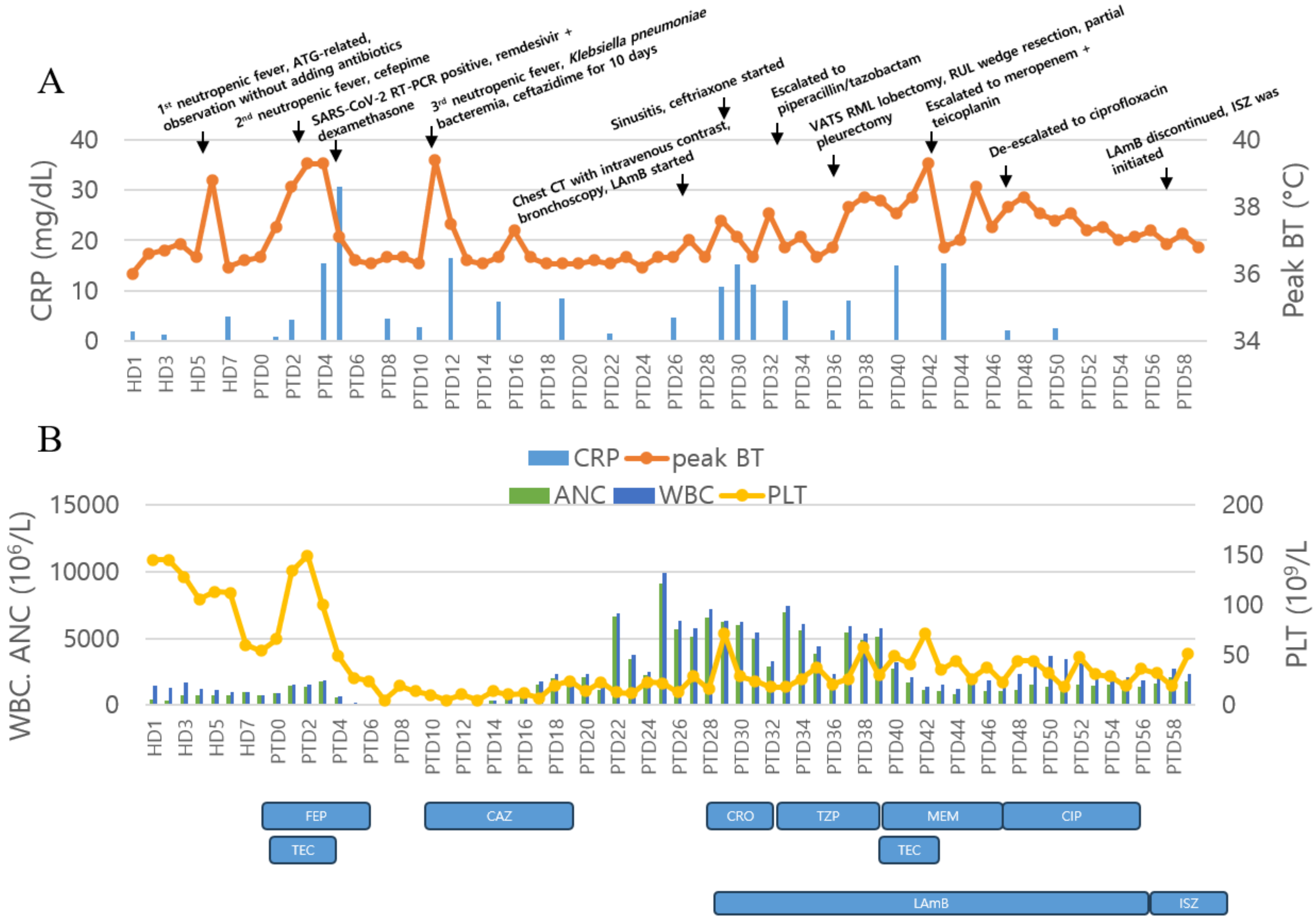

Clinical course of the patient from admission to discharge. (A) Timeline of key events alongside trends in body temperature and C-reactive protein levels. (B) Changes in peripheral blood cell counts and periods of antibiotics administration. ATG, anti-thymocyte globulin; ANC, absolute neutrophil count; BT, body temperature; CAZ, ceftazidime; CIP, ciprofloxacin; CRO, ceftriaxone; CRP, C-reactive protein; CT, computed tomography; FEP, cefepime; HD, hospital day; ISZ, isavuconazole; LAmB, liposomal amphotericin B; MEM, meropenem; PLT, platelet count; PTD, post-transplant day; RML, right middle lobe; RT-PCR, reverse transcription-polymerase chain reaction; RUL, right upper lobe; SARS-CoV-2, severe acute respiratory syndrome coronavirus 2; TEC, teicoplanin; TZP, piperacillin/tazobactam; VATS, video-assisted thoracic surgery; WBC, white blood cell count.
