# Supplementary material for: Pathologically Confirmed Dual Coronavirus Disease 2019-Associated Tracheobronchial Aspergillosis and Pulmonary Mucormycosis in a Non-Endemic Region: A Case Report
Source: J Clin Med. 2025 Aug 5;14(15):5526. doi: 10.3390/jcm14155526 (PMC12347539; doi:10.3390/jcm14155526)
Supplement: Supplementary file 1 [file jcm-14-05526-s001.zip › jcm-3751502-supplementary/Supplementary_materials_S2_CAREchecklists.pdf]

CARE Checklist (2013) of information to include when writing a case report

(cc) BY-NC-ND

Topic	Item	Checklist item description	Reported on Page
**Title**	**1**	The words "case report" should be in the title along with the area of focus	Page 1, line 2-3
**Key Words**	**2**	2 to 5 key words that identify areas covered in this case report	Page 3, line 47-48
**Abstract**	**3a**	Introduction—What is unique about this case? What does it add to the medical literature?	Page 2, line 23-29
	**3b**	The main symptoms of the patient and the important clinical findings	Page 2, line 30-40
	**3c**	The main diagnoses, therapeutics interventions, and outcomes	Page 2, line 30-40
	**3d**	Conclusion—What are the main "take-away" lessons from this case?	Page 2, line 41-45
**Introduction**	**4**	One or two paragraphs summarizing why this case is unique with references	Page 4, line 59-71
**Patient Information**	**5a**	Demographic information and other patient specific information	Page 4-5, line 74-77
	**5b**	Main concerns and symptoms of the patient	Page 4-5, line 74-87
	**5c**	Medical, family, and psychosocial history including relevant genetic information (also see timeline).	Page 4-5, line 74-77
	**5d**	Relevant past interventions and their outcomes	Page 4-5, line 74-85
**Clinical Findings**	**6**	Describe the relevant physical examination (PE) and other significant clinical findings	Page 4-5, line 74-80
**Timeline**	**7**	Important information from the patient's history organized as a timeline	Page 4-5, line 74-85
**Diagnostic Assessment**	**8a**	Diagnostic methods (such as PE, laboratory testing, imaging, surveys)	Page 5-6, line 86-112
	**8b**	Diagnostic challenges (such as access, financial, or cultural)	N[a]
	**8c**	Diagnostic reasoning including other diagnoses considered	Page 5-6, line 91-112
	**8d**	Prognostic characteristics (such as staging in oncology) where applicable	Page 4-5, line 74-85
**Therapeutic Intervention**	**9a**	Types of intervention (such as pharmacologic, surgical, preventive, self-care)	Page 5-6, line 99-116
	**9b**	Administration of intervention (such as dosage, strength, duration)	Page 6, line 104-116
	**9c**	Changes in intervention (with rationale)	Page 5-6, line 96-116
**Follow-up and Outcomes**	**10a**	Clinician and patient-assessed outcomes (when appropriate)	Page 6, line 113-116[b]
	**10b**	Important follow-up diagnostic and other test results	N[c]
	**10c**	Intervention adherence and tolerability (How was this assessed?)	N[d]
	**10d**	Adverse and unanticipated events	N[e]
**Discussion**	**11a**	Discussion of the strengths and limitations in your approach to this case	Page 7, line 143-146

	**11b**	Discussion of the relevant medical literature	Page 7, line 127-142
	**11c**	The rationale for conclusions (including assessment of possible causes)	Page 7-8, line 143-159
	**11d**	The primary "take-away" lessons of this case report	Page 8, line 160-167
**Patient Perspective**	**12**	When appropriate the patient should share their perspective on the treatments they received	N[f]
**Informed Consent**	**13**	Did the patient give informed consent? Please provide if requested	**Yes**

[a] No specific diagnostic barrier encountered.

[b] Patient-assessed outcome was not reported.

[c] The patient was reported to be clinically stable at discharge; no further follow-up test results were reported.

[d] Intervention adherence not formally assessed; tolerability inferred from absence of reported adverse events during therapy.

[e] No adverse or unanticipated events were documented during antifungal therapy or post-surgical recovery.

[f] Patient perspective was not obtained at the time of documentation.
